# Lugol’s solution but not formaldehyde affects bone microstructure and bone mineral density parameters at the insertion site of the rotator cuff in rats

**DOI:** 10.1186/s13018-021-02394-6

**Published:** 2021-04-13

**Authors:** Xaver Feichtinger, Patrick Heimel, Claudia Keibl, David Hercher, Jakob Emanuel Schanda, Roland Kocijan, Heinz Redl, Johannes Grillari, Christian Fialka, Rainer Mittermayr

**Affiliations:** 1 Ludwig Boltzmann Institute for Experimental and Clinical Traumatology, Vienna, Austria; 2 AUVA Trauma Center Vienna - Meidling, Vienna, Austria; 3 Department of Orthopedic Surgery II, Herz-Jesu Hospital, Vienna, Austria; 4 Austrian Cluster for Tissue Regeneration, Vienna, Austria; 5 Karl Donath Laboratory for Hard Tissue and Biomaterial Research, Department of Oral Surgery, University Clinic of Dentistry, Medical University of Vienna, Vienna, Austria; 6 Ludwig Boltzmann Institute of Osteology, 1st Medical Department at Hanusch Hospital, Vienna, Austria; 7 Center for the Musculoskeletal System, Medical Faculty, Sigmund Freud University, Vienna, Austria

**Keywords:** Rotator cuff tear, Bone-tendon interface, Lugol, Formaldehyde, MicroCT

## Abstract

**Background:**

This study aimed to investigate whether rodent shoulder specimens fixed in formaldehyde for histological and histomorphometric investigations and specimens stained using Lugol’s solution for soft tissue visualization by micro-computed tomography (microCT) are still eligible to be used for bone architecture analysis by microCT.

**Methods:**

In this controlled laboratory study, 11 male Sprague-Dawley rats were used. After sacrifice and exarticulation both shoulders of healthy rats were assigned into three groups: (A) control group (*n* = 2); (B) formaldehyde group (*n* = 4); (C) Lugol group (*n* = 5). Half of the specimens of groups B and C were placed in a 4% buffered formaldehyde or Lugol’s solution for 24 h, whereas the contralateral sides and all specimens of group A were stored without any additives. MicroCT of both sides performed in all specimens focused on bone mineral density (BMD) and bone microstructure parameters.

**Results:**

BMD measurements revealed higher values in specimens after placement in Lugol’s solution (*p* < 0.05). Bone microstructure analyses showed increased BV/TV and Tb.Th values in group C (*p* < 0.05). Specimens of group C resulted in clearly decreased Tb.Sp values (*p* < 0.05) in comparison to the control group. Formaldehyde fixation showed minimally altered BMD and bone microstructure measurements without reaching any significance.

**Conclusions:**

MicroCT scans of bone structures are recommended to be conducted natively and immediately after euthanizing rats. MicroCT scans of formaldehyde-fixed specimens must be performed with caution due to a possible slight shift of absolute values of BMD and bone microstructure. Bone analysis of specimens stained by Lugol’s solution cannot be recommended.

## Introduction

Depending on tear size, healing failure rates of rotator cuff reconstructions are reported up to 94% [1, 2]. The reasons for the high failure rate are multifactorial. Bony changes at the tendon insertion site in patients suffering from rotator cuff tears have been previously described [3–5]. Decreased bone mineral density and diminished bone microstructure were shown to have an important influence on the risk of re-ruptures [6, 7]. Characteristics of the reconstructed tendon itself, namely its organization and structure in addition to muscle quality are important factors to consider [8].

Experimental animal models have been developed to investigate additional treatment possibilities to avoid these problems associated with re-tears [9, 10].

As bone, tendon, and muscle structures constitute a functional unit, simultaneous investigations of these structures in one sample would be of great interest. To investigate bone structure and bone mineral density (BMD), specimens are routinely placed in saline solution, 4% paraformaldehyde, or are frozen natively at − 80 °C until micro-computed tomography (microCT) testing [8, 11, 12].

Recently, a staining method for soft tissue visualization (muscle and tendon structures as well as nervous tissue) by microCT analysis was described [13–15]. Thereby, Lugol’s solution, defined as a mixture of two parts potassium iodide in water and one part iodine, is used for staining of specimens [13, 16, 17]. Until now, little is known about the behavior of Lugol’s solution on bone structure parameters. To histologically investigate tendon and muscle structures, 4% buffered formaldehyde solution is commonly used for fixation [18]. Afterwards, different staining methods are performed to visualize the respective structures of interest.

Consequently, the aim of this study was to investigate the influence of Lugol’s solution and formaldehyde solution on bone microstructure and BMD during microCT analysis, as they may have a profound influence in interpreting microCT results in biomechanical rotator cuff studies.

The primary hypothesis was that Lugol’s solution has no influence on bone microstructure and BMD in microCT analysis compared to native scans.

Secondly, we hypothesized that 4% buffered formaldehyde solution has no influence on bone microarchitecture using microCT analysis compared to native scans.

## Methods

The study was authorized by the Institutional Animal Care and Use Committee (No. 504113/2016/16). Eleven male Sprague-Dawley rats were used for this study. Weight and age of the rats were homogeneous in the 3 groups (390–410 g). The rats (2/cage) were housed in a light- and temperature-controlled room. After 1 week of acclimatization period, rats were euthanized under deep anesthesia by an intracardially overdose of thiopental. Subsequently, both humeri were exarticulated. Supraspinatus tendon and muscle structures were preserved including the enthesis. Rats were divided into three groups: (A) control group (*n* = 2); (B) formaldehyde group (*n* = 4); (C) Lugol group (*n* = 5). MicroCT scans of the control group were performed immediately after exarticulation. Right shoulders of the Lugol group were placed in Lugol’s solution for 24 h at 4 °C [13, 16, 17]. Right shoulders from the formaldehyde group were placed in 4% buffered formaldehyde solution for 24 h at 4 °C. After 24 h, microCT scans of the Lugol and formaldehyde group were performed in the residual staining solution. The contralateral shoulders were scanned natively without any additional substances and were compared to the corresponding staining method within the group. MicroCT (μCT 50, SCANCO Medical AG, Brüttisellen, Switzerland) scanning and segmentation was conducted by a blinded examiner [19]. The specimens were placed in 15 ml centrifugation tubes. MicroCT scans were conducted at 200 μA, 90 kVp with a field-of-view of 20.48 mm and reconstructed to a resolution of 10 μm. The SCANCO calibration phantom was used for calibration.

**Fig. 1:**
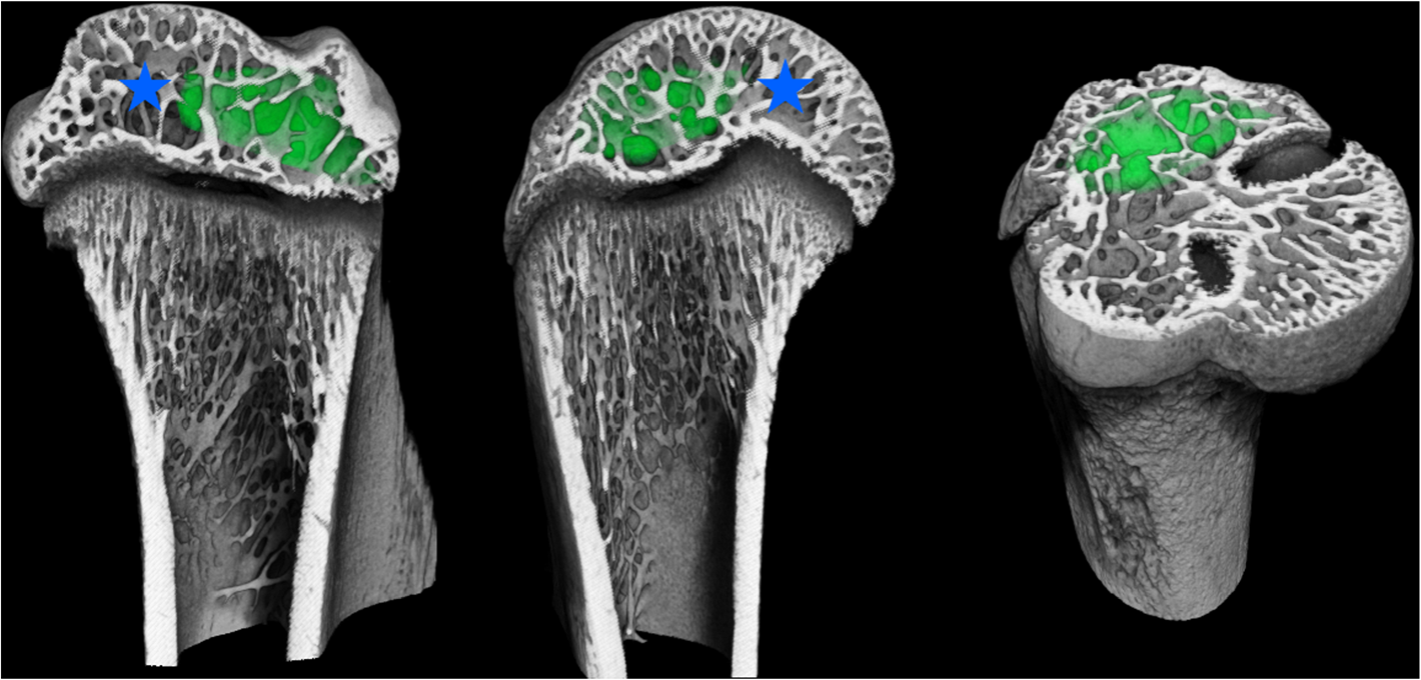
Computer tomography analysis in sagittal plane (left), frontal plane (middle), and transverse plane (right). The epiphysis is marked with a blue star. Green area marks subvolume of the epiphysis space at tendon attachment as region of interest (ROI)

Fiji was used to transform the humeri to an upright orientation along the *Z* axis [20, 21]. The scans were then rigidly registered using Amira 6.2 (FEI, Thermo Fisher Scientific, Hillsboro, OR, USA) to a single specimen. Regions of interest were manually drawn in the trabecular space of the epiphysis equally in all groups. A sub-volume of the epiphysis space at tendon attachment site was manually selected in all specimens as region of interest (ROI) (Fig. 1). To prevent measurement errors due to differences in the geometry of the specimen, the regions were then corrected using Definiens Developer XD 2.0 (Definiens AG, Munich, Germany). In Definiens, a ruleset was developed to segment the cortical portion of the specimen, based on local volumetric bone density. The cortical portion was not considered for measurement. Two measurements were performed with different thresholds for bone segmentation. Firstly, a measurement using a fixed threshold of 500 mgHA/cm^3^ for all native and formalin-stained specimen. Secondly, due to obvious differences in brightness in the Lugol stained specimen, a measurement was performed using a threshold calculated via Otsu’s automatic threshold selection in the ROI of each Lugol stained specimen and their native contralateral controls [22]. Affected and contralateral control were always thresholded and measured with the same method.

Bone mineral density and bone volume fraction were measured in Definiens. For measurement of bone mineral density, the outer 20 μm of bone structures were excluded. The segmented trabecular structures were exported as binary image stacks and analyzed in Fiji using the BoneJ plugin for measurement of trabecular thickness and spacing [23].

BMD (mgHA/cm^3^) as well as bone microstructure parameters including mean bone volume fraction (BV/TV; %), mean trabecular thickness (Tb.Th, μm), and mean trabecular spacing (Tb.Sp, μm) were then calculated. Analyses of the ipsilateral and contralateral side were then compared and a ratio was generated for statistical analyses.

### Statistical analyses

Testing for normal distribution was conducted using the D’Agostino and Pearson omnibus normality test. Accordingly, Kruskal-Wallis test and Mann-Whitney test were performed. In graphs, values are presented as mean values and associated standard error of mean. For calculation, a ratio of the affected to the non-affected native side for each parameter was created. A *p* value < 0.05 was considered statistically significant. GraphPad Prism version 8.3.1 (GraphPad Software, La Jolla, CA, USA) was used for statistical calculations.

## Results

### Bone mineral density (BMD)

**Fig. 2:**
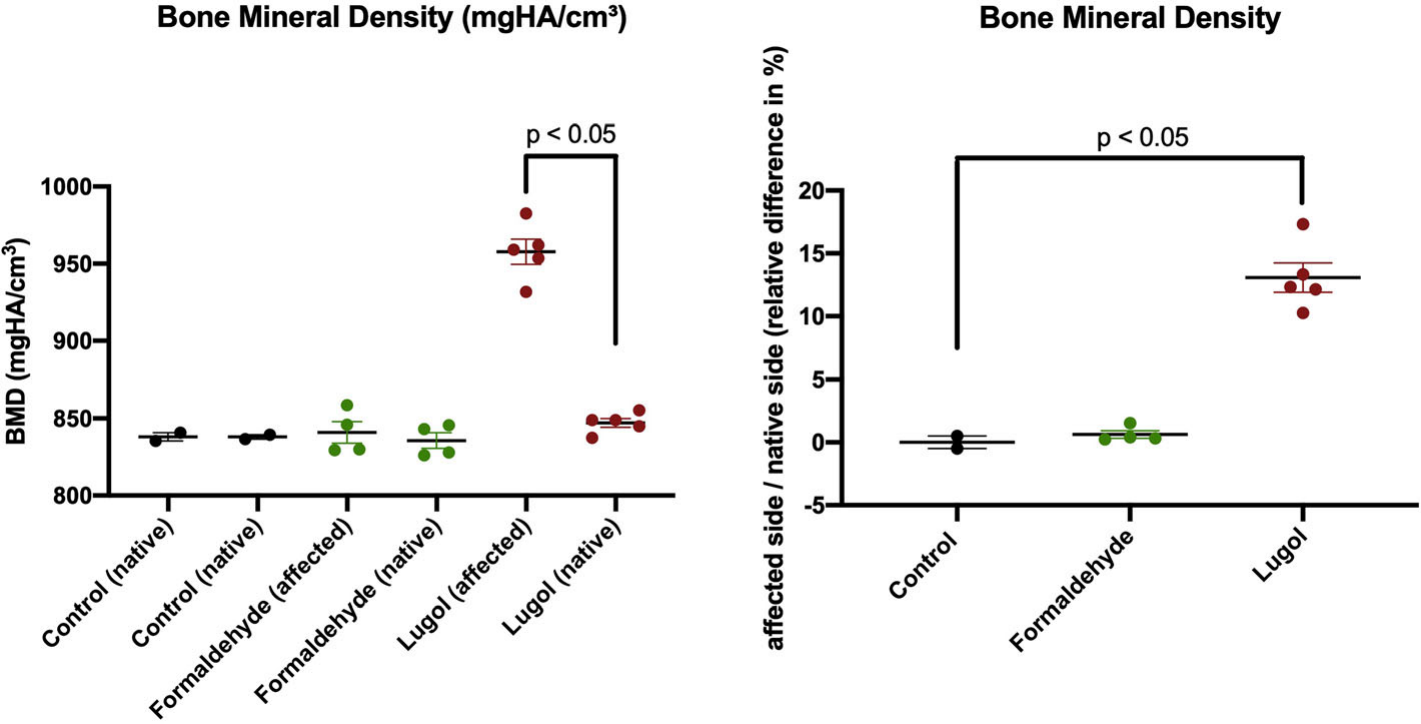
Bone mineral density (BMD): *left:* absolute values. *right:* relative difference (%) of affected side and native contralateral side. Values are presented as mean values and associated standard error of mean

BMD measurements revealed higher values in specimens after storage in Lugol’s solution (Fig. 2). Analyses showed significantly higher (*p* < 0.05) ratios of the affected side compared to the native contralateral side within group C. Group B revealed minimally increased ratios in comparison to the control group without reaching significance (Fig. 2).

### Bone microarchitecture

**Fig. 3:**
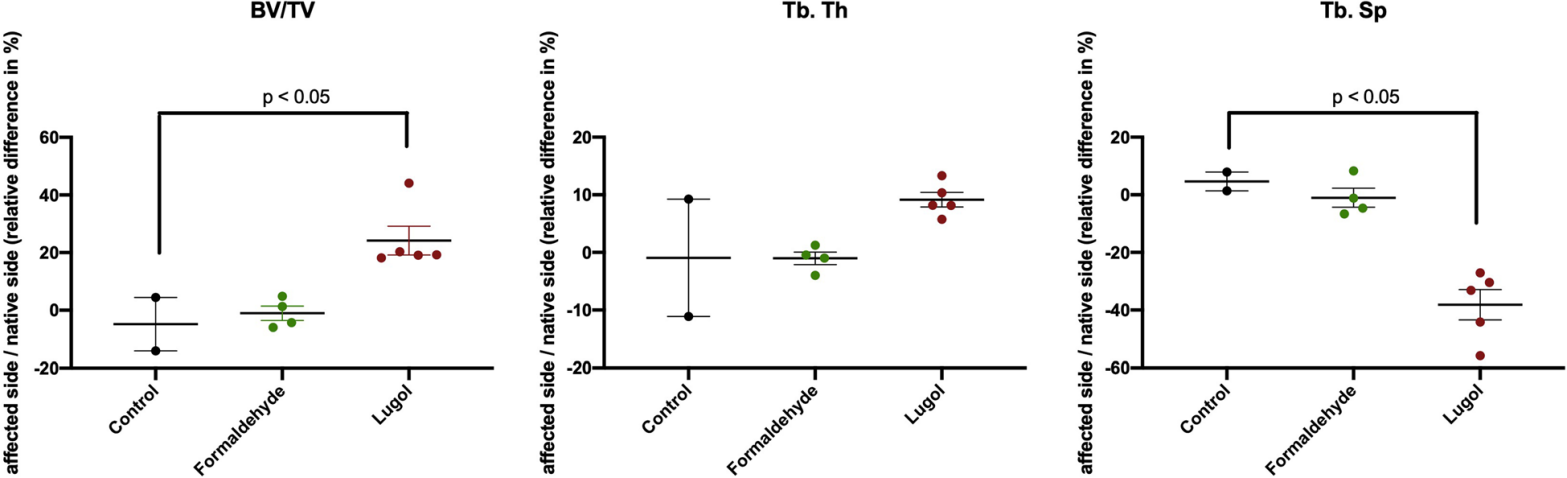
Bone microstructure: relative difference (%) for bone volume fraction (BV/TV); trabecular thickness (Tb.Th); trabecular spacing (Tb.Sp). Values are presented as mean values and associated standard error of mean

Shown are the relative differences between the affected and native contralateral control as a ratio affected/native. Bone microstructure analyses showed increased BV/TV and Tb.Th ratios in group C (*p* < 0.05) (Fig. 3). Specimens from group C resulted in significantly decreased (*p* < 0.05) Tb.Sp ratios compared to the control group (Fig. 3). Formaldehyde fixation showed minimally increased BV/TV and Tb.Th ratios and slightly decreased Tb.Sp measurements without reaching significance.

## Discussion

This study aimed to investigate the influence of formaldehyde fixation and staining using Lugol’s solution on BMD and bone microstructure of the rotator cuff insertion site in rats. Results showed significantly altered values after immersion in Lugol’s solution for 24 h. Comparisons of bone parameters between formaldehyde-fixed specimens and native controls did not differ.

The importance of bone structures in rotator cuff tears and their influence on success after reconstruction was shown in earlier studies. Meyer et al. described significant differences regarding BMD in human cadavers with rotator cuff tears in comparison to their contralateral side without tears [4]. Oh et al. described seven different regions of bone density in the proximal humerus in patients with unilateral rotator cuff repairs stating in the posterolateral portion the highest volumetric BMD [5]. Chung et al. presented the influence on success of reconstruction and stated that lower BMD and fatty infiltration are associated with lower healing results after reconstruction [6]. Also bone microstructure was deteriorated in patients with rotator cuff tears. Kirchhoff et al. showed differences in BV/TV values at the proximal humerus by high-resolution quantitative computed tomography [7]. These findings underline the importance of bone structure evaluation in rotator cuff tear models and require further investigations of treatment options for improvement of bone deficiencies after rotator cuff tears [10, 24].

Not only bone quality evaluated by microCT investigations are of high scientific interest. Also histomorphometric analysis of bone, tendon, and muscle are essential methods of analysis [25, 26]. Myocellular and intramuscular fat infiltration, atrophy, and fibrosis of muscle and tendon structures are associated with high rates of healing failure [25, 27]. Kim et al. stated that tears at the anterior part of the supraspinatus tendon particularly need to be treated early due to the high risk of fatty infiltration associated with inferior clinical outcome [27].

These findings require techniques that allow for investigations in both bone structures and soft tissue structures at a high-quality level. Until now, it was unclear if specimens fixed with formaldehyde for histological and histomorphometric investigations or specimens stained by Lugol’s solution can be used for reliable bone structure evaluations by microCT as well.

**Fig. 4:**
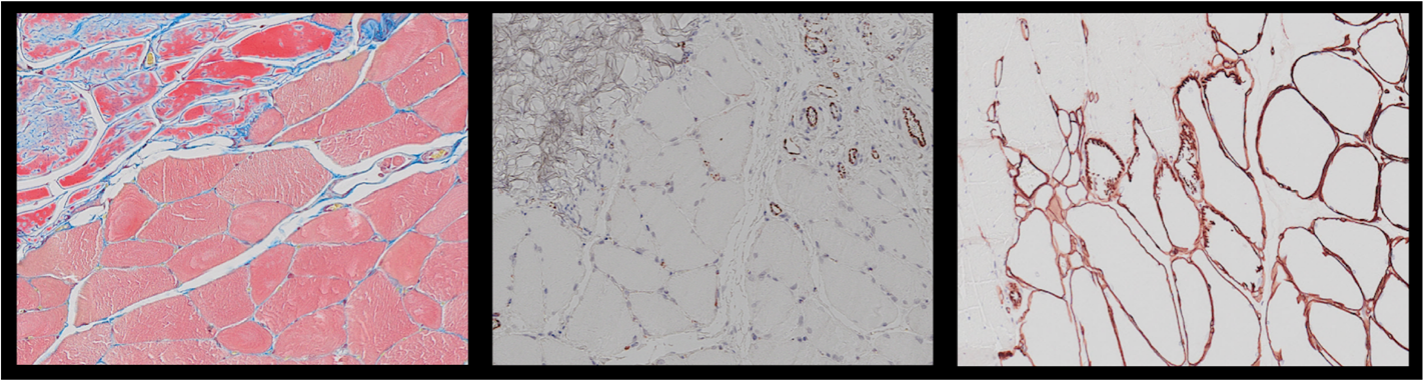
Stained sections of musculo-tendinous transition zones: l*eft:* Martius, Scarlet, and Blue (MSB) for muscle and tendon quality assessment, *middle:* CD31 stained section for vascularization analysis, *right:* collagen III stained section for muscle and tendon regeneration analysis

For histological investigation of soft tissue structures, a 4% buffered formaldehyde solution is commonly used for fixation [18]. Afterwards, different staining methods are used to investigate structures of interest [9]. Particularly in rotator cuff reconstruction animal models, investigations of the musculo-tendinous transition zone including vascularization and collagen types relations (Fig. 4) are of great interest. In this study, microCT scans of shoulders from the formaldehyde group showed minimally increased ratios in comparison to the control group without reaching significance for BMD measurements. Bone microarchitecture assessment showed in the formaldehyde group minimally increased BV/TV and Tb.Th ratios and slightly decreased Tb.Sp measurements without reaching significance. Earlier studies showed the effect of formaldehyde on the biomechanical properties of bone [28, 29]. Elements in the hydroxyapatite of bone as Ca, P, and Mg, can dissolve in formaldehyde and alter the biomechanics [28, 29]. In the present study, the minimal changes of BMD and microarchitecture parameters in the formaldehyde group reflect ongoing processes of the bone but minimize its relevance for microCT analyses. The duration of preservation in formaldehyde seems to play an important role, as other studies investigated biomechanics after long-term preservation, in this study shoulders were placed in 4% buffered formaldehyde solution for 24 h. Due to minimal changes after formaldehyde preservation in microCT analyses in this study, native scans are recommended to avoid incorrect measurements.

**Fig. 5:**
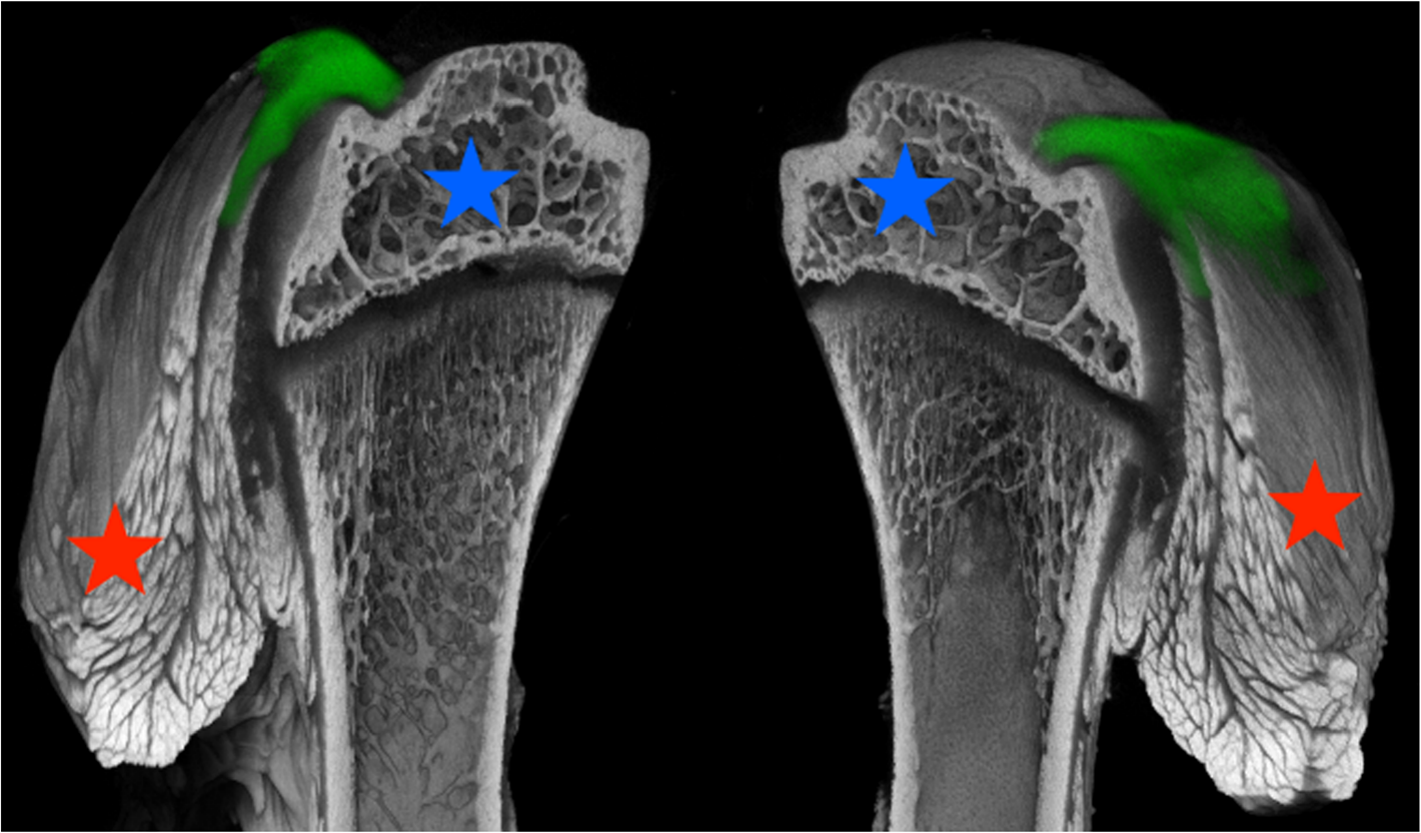
Sagittal cut of a humerus specimen in Lugol’s solution. Blue star: epiphysis. Red star: supraspinatus muscle. Green area marks supraspinatus tendon structure

Recent studies describe staining methods with Lugol’s solution enabling visualization and analyses of soft tissues such as muscle and tendon structures as well as nerve structures by microCT analysis [13, 14, 16, 17, 30] (Fig. 5). Consequently, 3D reconstructions of soft tissue structures including nerve visualization enable new outcome measurement options in experimental studies. The importance of neurologic deficiencies have been shown earlier and are of high scientific interest in experimental rotator cuff tear animal models [31, 32].

In this study, significantly higher BMD values were measured in specimens after storage in Lugol’s solution. Bone microarchitecture evaluation resulted in clearly increased BV/TV and Tb.Th ratios as well as decreased Tb.Sp measurements after preservation in Lugol’s solution. Despite rigidity and the low permeability of bone tissue, the effect of Lugol’s solution showed significant changes in microCT analysis. The effect most probably depends on time of staining, as the acidity changes during staining with Lugol’s solution and the pH begins to decrease. As a result of the lowered pH, decalcification of bone increases similarly to other acidic stains [13]. In the present study, the main interest was in soft tissues on the outer surface of the specimen. The specimens were thus only stained for a relatively short time, resulting in little stain reaching the trabecular space of the epiphysis. Despite this minimal staining, the effect on the bone measurements was significant. If soft tissues in the trabecular space of the epiphysis or medullary cavity are of interest, longer staining durations and a higher amount of stain are necessary, compounding the negative effect on bone measurements. The exact amount of stain taken up by the tissue is difficult to control, resulting in a degree of variability in the final attenuation [13]. Measurements performed using a fixed threshold may therefore show differences between samples based on the individual staining. We attempted to correct for this using Otsu’s automatic threshold selection in the region of interest.

In conclusion, this study describes significant BMD and bone microstructure deteriorations in microCT analysis after staining of proximal humeri in Lugol’s solution. Formaldehyde fixation may have a slight influence on bone evaluation values in comparison to native microCT scans. Consequently, microCT scans of bone structures are recommended to be conducted natively and immediately after sacrifice of rats. MicroCT scans of formaldehyde-fixed specimens can be performed with caution of interpretation due to a possible shift of absolute values of BMD and bone structure. Bone analyses of specimens stained by Lugol’s solution are not recommended due to significant deteriorations.

## Data Availability

The datasets used and analyzed during the current study are available from the corresponding author on reasonable request.

## Abbreviations

microCT: Micro-computed tomography
BMD: Bone mineral density
BV/TV: Mean bone volume fraction
Tb.Th: Mean trabecular thickness
Tb.Sp: Mean trabecular spacing
